# Nucleic acids and analogs for bone regeneration

**DOI:** 10.1038/s41413-018-0042-7

**Published:** 2018-12-27

**Authors:** Yuxin Zhang, Wenjuan Ma, Yuxi Zhan, Chenchen Mao, Xiaoru Shao, Xueping Xie, Xiawei Wei, Yunfeng Lin

**Affiliations:** 1 State Key Laboratory of Oral Diseases, National Clinical Research Center for Oral Diseases, Department of Oral Surgery, West China Hospital of Stomatology, Sichuan University, Chengdu, 610041 People’s Republic of China; 2 Lab of Aging Research and Nanotoxicology, State Key Laboratory of Biotherapy, West China Hospital, Sichuan University, Chengdu, Sichuan 610041 People’s Republic of China

## Abstract

With the incidence of different bone diseases increasing, effective therapies are needed that coordinate a combination of various technologies and biological materials. Bone tissue engineering has also been considered as a promising strategy to repair various bone defects. Therefore, different biological materials that can promote stem cell proliferation, migration, and osteoblastic differentiation to accelerate bone tissue regeneration and repair have also become the focus of research in multiple fields. Stem cell therapy, biomaterial scaffolds, and biological growth factors have shown potential for bone tissue engineering; however, off-target effects and cytotoxicity have limited their clinical use. The application of nucleic acids (deoxyribonucleic acid or ribonucleic acid) and nucleic acid analogs (peptide nucleic acids or locked nucleic acids), which are designed based on foreign genes or with special structures, can be taken up by target cells to exert different effects such as modulating protein expression, replacing a missing gene, or targeting specific gens or proteins. Due to some drawbacks, nucleic acids and nucleic acid analogs are combined with various delivery systems to exert enhanced effects, but current studies of these molecules have not yet satisfied clinical requirements. In-depth studies of nucleic acid or nucleic acid analog delivery systems have been performed, with a particular focus on bone tissue regeneration and repair. In this review, we mainly introduce delivery systems for nucleic acids and nucleic acid analogs and their applications in bone repair and regeneration. At the same time, the application of conventional scaffold materials for the delivery of nucleic acids and nucleic acid analogs is also discussed.

## Bone Regeneration: Nucleic acids and analogs in bone repair

Used with an appropriate delivery system, nucleic acids and nucleic acid analogs have excellent potential for bone repair and regeneration. Owing to various challenges with bone tissue regeneration, current research is largely focused on gene therapy, which employs genes to treat or prevent disease, and such new materials as nucleic acids (DNA and RNA) and nucleic acid analogs (compounds structurally similar to naturally occurring nucleic acids). A team headed by Yunfeng Lin at Sichuan University, China conducted a review of delivery systems for nucleic acids and nucleic acid analogs and their application in bone repair and regeneration. The authors identified the use of biomaterial scaffolds (which mimic living tissue) as one of the most important research areas for gene therapy, and that strategy has proven effective with all types of bone regeneration and repair.

## Introduction

Many organ systems in humans possess the extraordinary potential to regenerate and repair. One of the largest organ systems in the human body is the bone tissue, which also exhibits spontaneous self-repair ability following injury. Thus, bone tissue is a dynamic organ that can be remodeled. Traditionally, this tissue has been considered a structural organ that can facilitate locomotion and provide protection for other vital organ systems.^1^Bone tissue is also a significant reservoir for various minerals including phosphate, calcium, and magnesium. Moreover, it contains organic molecules such as amorphous matrix and collagen fibers.^2^ Therefore, the health of this tissue and treatments for bone-associated diseases are vital for human health.

Various diseases can occur in bone tissue that seriously threaten patient quality of life. The main clinical manifestations of most bone-associated diseases, such osteoarthritis (OA), rheumatoid arthritis, and bone cancer, are bone-arthrosis pain and bone loss.^3–5^ The main direction and focus of therapeutic research is methods to promote bone tissue regeneration and reduce bone defects. The dysfunction or loss of bone tissue, which mainly result from inflammation, injury, trauma, diseases, ageing, or genetic predisposition, can lead to significant morbidity as well as various of socio-economic issues.^6^ Typically, bone injuries can be categorized into different subfields depending on the damaged areas as follows: maxillofacial, craniofacial, long bones, and spine. The most common sites of bone injury include the femur, shoulder, hip, wrist, tibia, and ankle, together with vertebral and maxilla- and cranio-facial injuries^2,7^. Bone loss can lead to poor quality of life; therefore, effective treatments are essential. When the extent of bone tissue injuries supersedes that of self-rehabilitation, spontaneous regeneration might not occur. Therefore, this might result in scar formation or non-union and even persistent bone defects.^8^ At present, traditional therapeutic methods for the regeneration of bone tissue mainly include autologous bone, allograft bone, and artificial bone grafting.^7^ However, each method is associated with some restrictions, which make it difficult to achieve different clinical requirements such as those related to infectious risks, rejection, and donor site morbidity. In addition, various methods for bone grafting and types of bone substitutes might be associated with potential risks and concerns for both patients or surgeons, which might need to be differentially addressed based on the specific patient. Hence, novel approaches are urgent to avoid these adverse issues and to treat bone defects.

In recent years, tissue engineering and regenerative medicine has developed as a multifaceted discipline combining various fields of bioengineering, materials science, pharmacology, medicine, and life sciences, with the same purpose to promote the regeneration of injured or diseased organs within the human body^9^. Bone tissue engineering also comprises a series of alternative techniques for the research and development of novel biological materials to avoid the disadvantages associated with traditional grafts. Main focuses in bone tissue engineering are stem cell therapy, biomaterial scaffolds, and biological growth factors. Stem cells used in cell therapy, which are subjected to long-term in vitro culture to produce sufficiently large quantities, are derived from the patients themselves to minimize the immune response.^10^Numerous studies have reported that transplanted stem cells might have poor viability and osteogenic differentiation potential, and different types of stem cells also have drawbacks and limitations.^11^ Biomaterial scaffolds are designed to imitate the extracellular matrix, which can provide the appropriate microenvironment for the growth of bone tissue by supporting and accelerating cell migration and facilitating osteogenic differentiation.^11,12^ Hence, biomaterial scaffolds should possess excellent biocompatibility, adaptive biodegradability, and hypo-immunogenicity. Nevertheless, the synthetic methods to produce different scaffolds are complicated, and biosafety also needs to be ameliorated. Biological growth factors, which exert functions that regulate cell proliferation, migration, and differentiation, have significant roles in bone tissue regeneration.^13^ However, biological growth factors require delivery systems to overcome their disadvantages including low stability and penetration levels.

Due to the many remaining challenges associated with bone tissue regeneration, current research mainly focuses on new materials such nucleic acids (deoxyribonucleic acid [DNA] and ribonucleic acid [RNA]) and nucleic acid analogs (peptide nucleic acids [PNA] and locked nucleic acids [LNA]).^14^These types of materials, having special structures or improved efficacy for gene therapy, might expand research directions and approaches for bone tissue regeneration. DNA and RNA, which exist in every living organism and are produced by natural biological processes, can also be artificially synthesized. Meanwhile, DNA and RNA can be degraded based on their biological properties, which mean that both possess excellent biodegradability and biocompatibility.^15^ However, there still are some defects associated with DNA and RNA, such as short half-life and unstable structures. PNA and LNA are nucleic acid analogs that can persist and function in the cell for an extended period of time.^16^ Regarding DNA, RNA, PNA, and LNA, they also have some drawbacks such as low transfection efficiency; therefore, some drug-carrier systems are utilized for such applications.^17^ Previous studies have shown that these molecules have enormous potential for bone tissue regeneration. In this review, we discuss the delivery systems for nucleic acids and nucleic acid analogs and their applications in bone repair and regeneration.

## Gene therapy strategy for bone tissue engineering

**Fig. 1:**
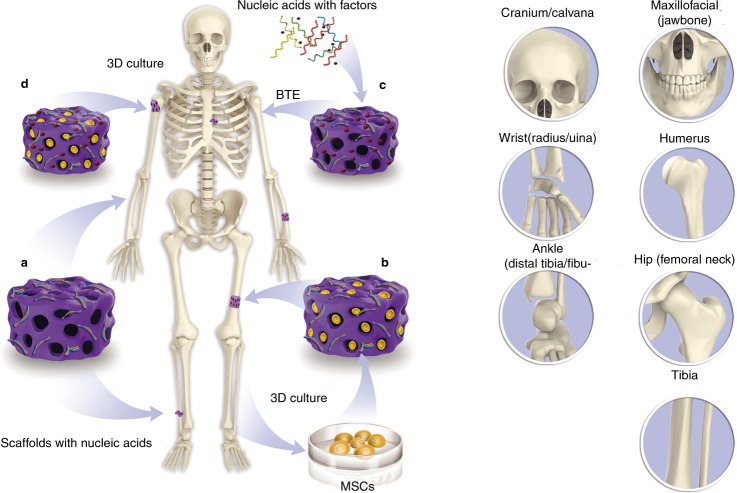
Main methods and essentials procedures that compose new bone tissue engineering and the major bone injury sites in body where strategies using 3D scaffolds. **a** Scaffolds combined with functional nucleic acid sequences. **b** MSCs culture and 3D culture with scaffolds and functional nucleic acid sequences. **c** Nucleic acids as bridge to connect factors to scaffolds. **d** MSCs culture and 3D culture with scaffolds and functional nucleic acid sequences which combined with factors

Traditional bone tissue engineering uses simple biological materials as scaffolds to induce the surrounding tissue to repair different bone defects. Biomaterial scaffolds can provide a similar structure to native bone architecture for cells and can regulate cells behaviors (e.g., cell proliferation, migration, and differentiation), which can contribute to the repair and formation of bone tissue.^18^ However, pure biological material scaffolds still have some limitations, as they lack precise controllability. Subsequently, biomaterials were further developed to combine growth factors and achieve synergistic effects on bone formation. Growth factors have large molecular weights and complex structures, which when combined with scaffold materials become unstable; moreover, the efficiency of synthesizing various growth factors is low.^19^ Nucleic acid and nucleic acid analogs with regulatory effects might avoid these disadvantages. Therefore, such drugs, which are formed by the combination of nucleic acids and different bio-material scaffolds, have increased potential for applications (Fig. 1).

**Fig. 2:**
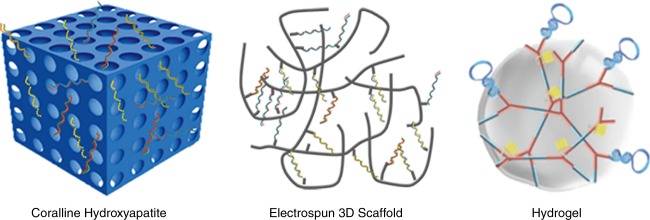
Traditional scaffolds for delivery of nucleic acids

Nucleic acid- and nucleic acid analog-based agents for bone tissue repair and regeneration can be classified as gene therapies for bone tissue engineering. Gene therapy consists of bio-material scaffolds, stem cells, and functional nucleic acid sequences.^20^ Biomaterial scaffolds can regulate cell behaviors and induce bone growth, and these are also referred to as extracellular matrices with osteoconductive, osteoinductive, and osteogenic characteristics. The most commonly-used bone repair scaffold materials have a demineralized bone matrix for bone conduction,^20^ coralline hydroxyapatite,^21,22^ Electrospun 3D Scaffolds (e.g., poly(3-hydroxybutyrate-co-4-hydroxybutyrate))^23,24^ and hydrogels.^19,25,26^ These scaffold materials have high-porosity, three-dimensional structures, and good biocompatibility, which can facilitate cell adhesion and the regulation of osteogenic-related genes. Due to the fact that these biological scaffolds are not associated with effective functions for bone tissue engineering, some previous studies have suggested that the application of pure nanocarriers to carry functional nucleic acid sequences can be used for the repair and regeneration of bone tissue (Fig. 2).

Seeded cells, which are autologous cells that are homologous to damaged bone tissue cells or pluripotent stem cells with differentiation potential, have also been considered as a promising treatment option for bone tissue engineering. The driving force for reconstructing new bone tissue is that the scaffold lacking seeded cells only has bone conduction potential.^27^ Therefore, stimulating new bone formation requires that seeded cells can differentiate via osteogenesis. For bone tissue engineering, optimal seeded cells should have the following characteristics: (I) broad-spectrum sources, simple material extraction, minimal damage to the body, and suitable for clinical applications; (II) strong proliferative potential in vitro and the ability to easily and stably express an osteoblast phenotype; (III) robust subculture ability; (IV) after being implanted into the body, they should adapt to the microenvironment of the damaged tissue and maintain osteogenic activity; (V) rapid osteogenesis with no carcinogenicity.^27^ Recent studies have shown that adipose-derived stem cells (ADSCs) and mesenchymal stem cells (MSCs) have promise for use in tissue repair due to their multi-directional differentiation abilities.^28–30^ MSCs are derived from the mesoderm during early embryonic development and are pluripotent stem cells that can differentiate into bone, cartilage, fat, and other cell types under specific induction conditions.^31–36^ As a subtype of MSCs, ADSCs are ideal stem cells for the regeneration of bone tissue. Compared to bone marrow-derived stem cells, ADSCs have become central to the field of bone tissue regeneration in recent years, because they are associated with rich sources, easy accessibility, and minimally invasive procedures for harvesting.^36–38^ In addition, enhancing the proliferation of autologous chondrocytes and fibroblasts is also an important target for bone tissue engineering. Inhibiting the formation and activity of osteoclasts has also been extensively studied.^39^ Further, the low survival rate and safety of seeded cells have also been one of the biggest challenges that has limited their clinical use. Therefore, functional nucleic acid sequences with enhanced safety have become a hot area of research over the past few years. The role of functional nucleic acid sequences is to enter cells and regulate the expression of osteogenesis-related genes, which is followed by the osteogenic induction of pure scaffold materials.

The repair and regeneration of bone tissue is a complex process, and many growth factors play an important role. Therefore, designing bio-scaffold materials with load-related growth factors is one important strategy in the field of bone tissue regeneration. Numerous studies have shown that bone morphogenetic proteins (BMPs) are the most important growth factors for the osteoinduction process, and can induce stem cells to differentiate into chondrocytes and osteoblasts. BMP-2, BMP-3, and BMP-7 are the most potent factors of the BMP family.^40–43^ These proteins are also members of the transforming growth factor-beta (TGF-β) superfamily. TGF-β is most abundant in bone and platelets, and its receptors are most expressed in osteoblasts. Studies have shown that TGF-β can not only regulate bone and chondrocyte growth and differentiation, but also regulate the expression and function of other growth factors during the repair and regeneration of cartilage.^44–46^ In addition, vascular endothelial growth factors, platelet-derived growth factor, and insulin-like growth factor, among others, are also associated with bone repair. However, for practical applications, these growth factors have some properties that make it difficult to directly connect them to biomaterial scaffolds; they also lack controllability.^47,48^ It is also difficult to transport these molecules directly to damaged areas and maintain long-term high concentrations. Therefore, a better option is to use gene therapy to regulate the expression of these growth factor-related genes or pathways to regulate the expression and function of growth factors in seeded cells. The expression of the relevant growth factors can be regulated by genetically manipulating seeded cells (i.e., gene therapy).^46^ This method is associated with solutions to many technical difficulties, such as reduced doses, precise delivery, and targeted release.^49^ Nucleic acid delivery, encompassing both DNA, RNA and nucleic acid analog therapeutics, within biomaterial scaffolds is the focus of the rest of this review.

**Fig. 3:**
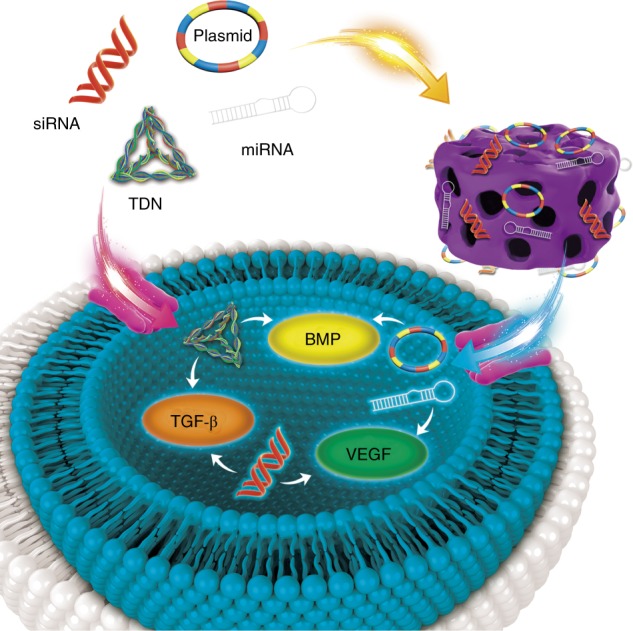
Target of nucleic acid following delivery to cells. pDNA, small-interfering RNA (siRNA) and microRNA(miRNA), TDNs, etc. can be delivered into cells by no-viral vector systems

In gene therapy for bone tissue engineering, delivery vectors deliver functional nucleic acid molecules into seed cells or enrich it around seed cells. Different nucleic acid molecules have different fates after entering the cell. For example, the plasmid DNA enters the cell and autonomously expresses the genetic information it carries.^50^ siRNA and miRNA bind to the corresponding gene target after entering the cell, inhibit the expression of related genes, and regulate cell behavior (Fig. 3).^51^

**Fig. 4:**
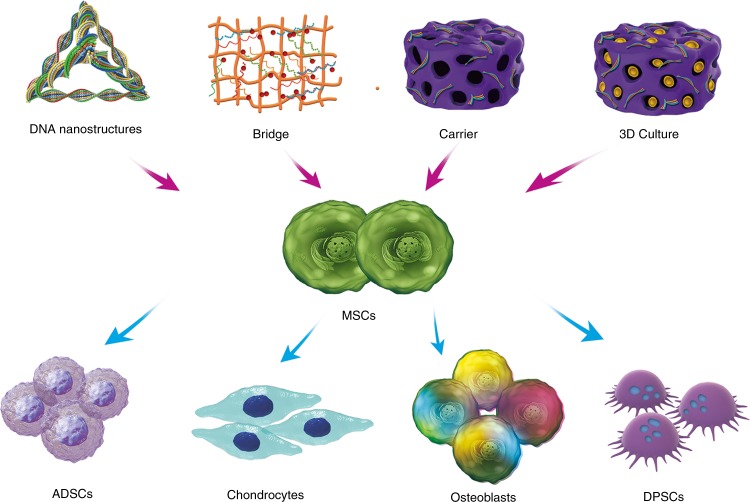
Different applications of functional nucleic acids in bone tissue engineering to promote the differentiation of MSCs

## Application of scaffold-based nucleic acid-based drugs in bone tissue engineering

Gene therapy essentially involves the introduction of specific signals into seed cells, regulating in the processing and secretion of synthesized and secreted a gene products (protein). The form of specific gene signals can be diverse, not only facilitating the transfer of the gene encoding the growth factor to the seed cell, but also a providing a specific sequence that regulates the expression of the growth factor. At present, the main problems in the field of gene therapy are effectiveness and safety. Since 1995, scientists in the field of gene therapy around the world have made great efforts to improve gene delivery systems and vectors; therefore, some new ideas, new technologies, and new methods have emerged. The first generation of vector design for gene therapy had obvious defects. In 1999, 18-year-old Jesse Gelsinger suffered from ornithine transcarbamylase deficiency after gene therapy trials due to the excessive intake of adenoviral vectors leading to multiple organ failure.^52^ With the progression of research, two major mainstream gene delivery vectors have emerged, specifically non-viral and viral vector systems. Non-viral vector systems have been extensively studied due to the insecurities associated with viral vector systems.^53–55^ Therefore, this review focuses on non-viral vectors that deliver nucleic acids and nucleic acid analogs (Fig. 4).

### Strategy for the application of biomaterial scaffolds loaded with DNA

Due to the special structure, physicochemical properties, and biological properties of DNA, DNA-based-nanomaterials can harbor remarkable features such as stability, flexibility, precise programmability, stimuli-responsive DNA conformations, and simple synthesis and modification.^14^ In bone tissue engineering, DNA materials have been widely used. Traditionally, genes encoding osteogenic growth factors can be transferred to seeded cells to promote efficient and stable expression in seeded cells (e.g., plasmid DNA).^50,56^ However, the delivery of plasmid DNA at the cellular level is very difficult, because of its particularly large molecular weight and negative charge, resulting in a low probability of cell internalization and susceptibility to degradation by nucleases. Hence, it is important to choose an adaptive carrier for the effective delivery of plasmid DNA. Studies have shown that plasmid DNA combined with hydrogels, without other transfection methods, can deliver plasmid DNA to seeded cells, and these structures have decreased cytotoxicity compared to that with other drugs.^57^ To promote alveolar bone regeneration, Yang and coworkers developed an injectable chitosan-based thermosensitive hydrogel scaffold (CS/CSn-GP) that incorporated BMP-2 plasmid DNA (pDNA-BMP2)-loaded chitosan nanoparticles (CS/CSn(pDNA-BMP2)-GP), and found that this material could effectively enhance new bone formation in calvarial defects of rats and accelerate bony defect healing in beagle dogs.^58^ DNA-based hydrogels can be coupled with biologically active substances that are difficult to deliver into mammalian cells or are easily degraded in vivo. However, biological scaffolds have specific functions and play a significant role in bone tissue to achieve bone regeneration and repair.^59,60^ In addition, DNA materials are also considered to be functional sequences that can be attached to biomaterial scaffolds, acting as a bridge for the binding of growth factors, functional proteins, nucleic acids, or nucleic acid analogs to biomaterial scaffolds.^53,61^ One end of the DNA molecule can be attached to the biomaterial scaffold, whereas the other end can be linked to a growth factor; this method solves the problems associated with low-level binding between large molecular proteins such as growth factors and biomaterial scaffolds.^62,63^ In researching osteoporosis, Ignatius and coworkers reported a biodegradable and biocompatible protein–DNA hybrid hydrogel carrying Rho-inhibiting C3 toxin for the targeted inhibition of osteoclast formation and activity.^19^ The functional protein was crosslinked via DNA hybridization without the application of reactive organic reagents or catalysts.

DNA material can be used not only as functional sequences to connect biomaterial scaffolds for the repair and regeneration of bone tissue, but also as a 3D nanostructure that can directly affect the osteogenic differentiation of seeded cells.^39,64–70^ Since the discovery of the principle of base pairing and the DNA double helical structure, interest in DNA has expanded beyond its genetic role to applications in nanotechnology and materials science. Studies have shown that based on Watson-Crick hybridization and special base sequence design, DNA can be assembled into nanostructures of different shapes and sizes. These DNA nanostructures, and especially tetrahedral DNA nanostructures (TDNs), have been shown to have good biocompatibility and have applications in many fields.^14^ Advantages have recently been uncovered for the application of TDNs to various types of stem cells including ADSCs, MSCs, and dental pulp stem cells (DPSCs). This has enhanced the possibility of repairing defective tissue, restoring damaged nerve tissue, and new therapeutic strategies for the regeneration of bone tissue such as alveolar bone. In addition, researchers found that these stem cells were important seeded cells that could be applied to the repair and regeneration of bone tissue.^67,71,72^

MSCs are derived from the mesoderm during early embryonic development and are pluripotent stem cells that can differentiate into bone, cartilage, fat, and other cell types under specific induction conditions.^31–36^ As a subtype of MSCs, ADSCs are ideal stem cells for the regeneration of bone tissue. Compared to bone marrow-derived stem cells, ADSCs have become central to the field of bone tissue regeneration in recent years, because they are associated with rich sources, easy accessibility, and minimally invasive procedures for harvesting.^36–38^ After treating ADSCs with 250nmolL^−1^ TDNs, some studies found that the gene and protein expression of *β-catenin*, *LEF-1*, and *Cyclin D* were upregulated, indicating that TDNs could induce osteogenic differentiation in ADSCs through the canonical Wnt/β-catenin signaling pathway. Numerous studies have also shown that the Wnt/β-catenin pathway is an important regulator of osteogenic differentiation in mesenchymal stem cells.^71,73–76^ β-catenin is a vital regulator of this pathway, and can enter the nucleus and interact with LEF-1. This latter protein in turn regulates the expression of Wnt-target genes (such as *Runx2*), ultimately promoting the osteogenic differentiation of MSCs.^29,37,77–79^ In addition, DPSCs existing in human dental pulp tissue have the potential for multi-directional differentiation (into another subtype of MSCs), and the resulting cells can be differentiated into multiple tissues such as teeth or bone with the induction of different cytokines. Hence, DPSCs have been considered a promising potential source of stem cells for bone tissue regeneration. Studies have shown that after treatment of dental pulp stem cells with 250nmolL^−1^ TDNs, dental pulp stem cells could proliferate significantly, the number of cells in S phase was increased, and the number of cells in G1 phase was decreased.^65,67^ In terms of osteogenic differentiation, the expression levels of genes and proteins related to osteogenesis (such as Runx2, Alp, OPN, and OCN) were also increased, which proves that this material plays an important role in inducing the osteogenic differentiation of DPSCs.^80–82^ The Notch signaling pathway is also an important regulator of DPSC osteogenic differentiation that can promote tooth formation, whereas Notch-1, Hes-1, and Hey-1 are key regulators of Notch signaling.^83^ After the addition of TDNs, the mRNA and protein expression levels of Notch1, Hes1, and Hey1 in DPSCs were significantly increased, and the Notch signaling pathway was activated, which also demonstrated that TDNs could induce the osteogenic differentiation of DPSCs into teeth by activating the Notch signaling pathway.^84–87^

Therefore, the pure TDNs have been shown to regulate the cellular behavior of various cells. For cells with differentiation potential, TDNs can be widely applied to bone regeneration through differentiation regulation. For terminally differentiated cells, maintenance and promotion of proliferation and migration make it practically important in the field of bone repair.^29,39,65^ Preliminary research in related fields shows that TDN has potential prospects in the field of bone repair and regeneration.

### Scaffold-based RNA delivery

In the past decades, the discovery and functional studies of non-coding small RNA molecules have changed people’s perception of RNA, and scholars were deepened the understanding and research of gene expression regulation.^88–91^ Small-interfering RNA (siRNA) and microRNA (miRNA) are two regulators of sequence-specific post-transcriptional gene expression and are the most important component of small RNAs. They regulate the expression of genes by mediating silencing mechanisms. Therefore, miRNAs and siRNAs are biomolecules with promising applications for drugs. When used as a therapeutic, both require a therapeutic effect by entering cells by means of a delivery vehicle.^88,92^

The size of miRNAs are endogenous small noncoding ~22-nt RNAs that recognize target mRNAs by interacting with recognition sites in 3ʹ-untranslated regions and subsequently post-transcriptionally repressing the expression of these genes.^93–95^ In bone tissue engineering, miRNAs also play important roles in processes that direct MSC fate, including cell proliferation, migration, osteogenesis, and chondrogenesis.^96^ Many miRNAs can regulate the osteogenic differentiation or angiogenesis of these seeded cells through different mechanisms. For example, activation of the classical Wnt signaling pathway plays a significant regulatory role during the formation of osteoblasts. Studies have found that miR-29a can further promote stem cell osteogenic differentiation by regulating the Wnt signaling pathway through a forward feedback loop.^97^ Whereas the classical Wnt signaling pathway induces miR-29 expression, this miRNA can also promote Wnt activity and osteogenic differentiation by targeting negative regulators of Wnt signaling including DKK1, Kremen, and sFRP2.^98,99^ Additionally, some studies have shown that the interaction between miR-34c and the PI3K-Akt signaling pathway induces a regulatory loop that is involved in the regulation of Vaspin during the osteogenic differentiation of mouse osteogenic precursor cells.^97,100,101^ It has been suggested that miRNA-based therapy has two directions. One is to use miRNA as a target and effectively prevent the binding of miRNAs to target genes by transferring a nucleotide sequence complementary to the target miRNA sequence (e.g., anti-miRNA).^102^ Inhibition of miRNA targeting can promote the expression of the target gene and protein. Another direction is the direct delivery of miRNA, which can further down-regulate target gene expression.^103,104^

Further, siRNA is also an effector molecule in the RNAi pathway. siRNA comprises a 21–23-bp short-segment, double-stranded RNA that specifically degrades mRNA with homologous sequences to inhibit expression of the target gene.^105^Therefore, siRNA is also an ideal therapeutic drug for bone tissue engineering applications. However, there are some obstacles for the practical application of miRNA and siRNA. For example, low transfection efficiency, poor targeting, and low stability in vivo make it difficult to achieve drug efficacy using miRNA.^106^ Further, the pharmacokinetic properties of siRNA are poor, which make it easy for these species to be biodegraded and also limit their clinical use. Therefore, it is necessary to find a suitable method to solve these problems.^51^ Currently, for the repair and regeneration of bone tissue, a bio-scaffold-based delivery strategy is mainly used.^105^ Some scholars directly embed naked miRNAs into biological scaffold materials to achieve the local, long-lasting, slow release of miRNAs, thereby facilitating a local miRNA sustained-release system that can target and maintain the expression of target genes in cell-free conditions. With this, the expected level of expression persists for a longer period of time, thereby effectively promoting bone regeneration. Previous studies have shown that miRNA-26a can promote bone regeneration through the positive regulation of angiogenesis–osteogenesis coupling.^107^ Li et al. developed a polymer with low toxicity (LP series) and one that was hyperbranched (HP series) as an miRNA transfection system, which was encapsulated in polylactic acid-glycolic acid sustained-release microspheres; the microspheres were further attached to a l-polylactic acid nano-porous scaffold to construct local miRNA.^102,107,108^ The sustained release system was degraded at different speeds with polylactic acid-glycolic acids of different molecular weights to achieve sustained release. In this study, miRNA-26a was embedded in this miRNA local sustained release system and implanted into a mouse skull defect model, and showed good ability to promote bone defect repair in situ. Based on the strategy of embedding small RNA into scaffold materials, some scholars have also studied the targeted modification of scaffold materials to selectively bind target cells and release embedded miRNA/siRNA. Treating bone-related diseases is of great significance. Studies have reported that polyurethane nanomicelles modified with an osteoblast-targeting peptide can be used to encapsulate miRNA/siRNA, which can be delivered for osteoblast release. Experimental results showed that this SDSSD-PU delivery system not only selectively targets the bone-forming surface, but also selectively targets osteoblasts without significant toxicity or the induction of an immune response. Anti-miR-214 was delivered to osteoblasts using the SDSSD-PU delivery system in this study, and results showed increased bone formation, improved bone microstructure, and increased bone mass in an ovariectomized osteoporosis mouse model.

For miRNA/siRNA-based gene therapy, there is a common problem, both of which are based on the small size of the RNA molecules. Due to the widespread existence of RNase, miRNAs and siRNAs are easily degraded during practical applications, and it is difficult to achieve expected results and effects. Therefore, additional chemical modification schemes are needed to prevent their degradation *in vivo*. There are currently three commonly used RNA modifications. The first is the modification of RNA molecules into a locked nucleotide acid (LNA) form, whereas the other is modification of RNA using 2-*O*-methoxyethyl phosphorothioate (2’-MOE). The third strategy is to modify RNA with cholesterol, which can promote phagocytosis by cells. Jessica E. Frith et al. studied miRNAs by regulating mTOR signalling to regulate human mesenchymal stem cells. In order to prolong the effect of miRNA, miRNA is modified to LNA. When a functional nucleic acid molecule is delivered, the nucleic acid molecules are modified into nucleic acid analogs or replaced with a nucleic acid analog, and a better inhibitory effect can be achieved.^95^ miR-29 family members are negative regulators of ECM synthesis, targeting mRNA encoding selected collagens and osteonectin/SPARC. Studies have reported that electrospun gelatin nanofibers can be used to embed miR-29a inhibitors (small chemically modified single-stranded hairpin oligonucleotides). The results showed that the miR-29a inhibitor was sustained for 72 h, allowing the inoculation to inhibit the expression of this molecule. Pre-osteoblasts on the agent-loaded nanofibers were found to synthesize more osteonectin, indicating the effective delivery of the inhibitor.

## Conclusion

The progress of bone tissue engineering is key to the treatment of various bone diseases. According to researchers, gene therapy is considered a modern therapeutic that can pave the way for the treatment of patients with bone diseases. Regarding gene therapy, the application of nucleic acids and nucleic acid analogs has been studied extensively. Due to some associated limitations of both approaches, the combination of nucleic acids/nucleic acid analogs with various biomaterial scaffolds has become a new research focus for gene therapeutics; this direction is currently being pursued and might be one of the most important research areas. This new type of treatment strategy has shown great potential in current research and has proven to be effective in all types of bone regeneration and repair. Meanwhile, this idea might be also applicable for the repair and regeneration of more complex organs and tissues such as nerve tissue and skin tissue. Therefore, this approach has marked potential for bone tissue engineering, which requires further investigations.

Scaffold-based nucleic acid drugs have shown great potential for the repair and regeneration of bone tissue. There are two main strategies. First, the functionalized nucleic acid sequence is first introduced into mesenchymal stem cells, and then the treated cells are combined with a suitable scaffold and implanted into the bone repair site. The implanted cells are used for production purposes. The protein source then induces differentiation in the target cell. These functionalized nucleic acid sequences can be growth factor-encoding genes (e.g., *BMP-2*, *BMP-3*, *BMP-7*, *TGF-β*, and *VEGF*, etc.), mediators that link scaffold materials and functional proteins, or 3D nanostructures that can regulate cell behavior (e.g., TDNs). Another approach is to use a composite delivery system containing a functionalized nucleic acid sequence that can directly target a specific tissue or organ. The functionalized sequence can be released by embedding it in the scaffold material and delivering it to the target site; it can also mediate further targeted modification of the scaffold material to enhance the accuracy of delivery of functionalized sequences. These sequences, including DNA, RNA, and nucleic acid analogs, can regulate seeded cells via different mechanisms to mediate the repair and regeneration of bone tissue. Studies on the small RNA-induced regulation of osteogenic differentiation have become a hot topic for bone tissue engineering, particularly in recent years.

Scaffold-based nucleic acid drugs have many advantages for gene transfer. Since they are not biological materials, they are non-immunogenic, do not cause an immune response, and are not cytotoxic. In contrast, most scaffold materials promote the osteogenic differentiation of seed cells. Combining the scaffold material with nucleic acids has a synergistic effect and enhances the utility of both. Therefore, this is the most important research direction for the repair and regeneration of bone tissue. In addition, due to the widespread presence of nucleases, nucleic acid drugs are susceptible to degradation. Therefore, finding ways to enhance the stability of nucleic acid drugs is also an urgent problem that needs to be solved. Although the combination of gene therapy and traditional bone tissue engineering methods represents a new treatment modality, its high efficiency, simplicity, speed, and specificity imply its great potential for application. Scaffold-based nanomedicine not only enhances the effectiveness of traditional methods, but also addresses biosafety concerns associated with other vectors. There have also been many exciting research results that are expected to be essential for the application of different treatments for bone tissue engineering.

Although important progress has been made in the field of gene therapy in recent years, the molecular mechanisms of different biomaterial scaffolds are still not exactly understood. With the expansion of this research, we have also found that there are still many problems to be solved for practical applications, such as controlling the expression level of growth factors and the timing of activation; no clear conclusions have been made regarding issues. In addition, the technologies association with nucleic acid and nucleic acid analog technologies are still not perfect. For example, DNA readily forms a polymer, RNA is degraded easily, and the synthesis of nucleic acid analogs can be inconsistent. Furthermore, the methods used to connect nucleic acids/nucleic acid analogs to biomaterial scaffolds need to be improved to increase the synthesis rate of composite materials. Therefore, this field awaits further exploration to enhance bone tissue regeneration and repair.
